# Building resilience from the start: integrating wellbeing and coping skills into psychiatric training

**DOI:** 10.1192/j.eurpsy.2026.10264

**Published:** 2026-07-31

**Authors:** S. M. M. Toparlak

**Affiliations:** CAMHS, Oxford Health NHS FT, Oxford, United Kingdom

## Abstract

**Abstract:**

Burnout among early-career psychiatrists is increasingly recognised as a significant threat to workforce sustainability, patient safety, and professional fulfilment. Long working hours, emotional intensity, and systemic pressures often converge during training, before clinicians have developed structured coping strategies. While institutional reform is essential, resilience-building within training programmes offers a powerful complementary approach.

This talk explores how wellbeing and coping skills can be proactively integrated into psychiatric training from the outset, rather than introduced reactively once distress emerges. Drawing on evidence from medical education, occupational psychology, and reflective practice initiatives including insights from the Bucks Movie Club article, which highlighted the value of narrative, shared reflection, and creative spaces in supporting clinician wellbeing-the session will examine practical strategies that can be embedded within training curricula.

Key areas include emotional regulation skills, structured reflective practice, peer-support forums, psychologically safe supervision models, and boundary-setting within high-demand clinical environments. The role of mentorship and facilitated debriefing following challenging clinical encounters will also be discussed. In addition, cognitive-behavioural principles, mindfulness-based approaches, and values-driven career planning will be considered as tools to strengthen professional identity and reduce vulnerability to burnout.

Resilience will be framed not as individual toughness, but as a dynamic process shaped by organisational culture, leadership, and team cohesion. The session will highlight how institutions can foster environments that normalise help-seeking, encourage creative and reflective engagement such as film-based discussion groups and equip trainees with sustainable coping tools early in their careers.

By shifting the focus from crisis response to early prevention, psychiatric training programmes can cultivate clinicians who are not only clinically competent but psychologically equipped to thrive. Integrating structured wellbeing education into training is not an optional enhancement, it is an investment in the future of the profession.

**Disclosure of Interest:**

None Declared

